# What is next in African neuroscience?

**DOI:** 10.7554/eLife.80488

**Published:** 2022-06-22

**Authors:** Kirsten A Donald, Mahmoud Maina, Nilesh Patel, Carine Nguemeni, Wael Mohammed, Amina Abubakar, Matthew Brown, Raliza Stoyanova, Andrew Welchman, Natasha Walker, Alexis Willett, Symon M Kariuki, Anthony Figaji, Dan J Stein, Amadi O Ihunwo, William Daniels, Charles R Newton

**Affiliations:** 1 University of Cape Town Cape Town South Africa; 2 University of Sussex Falmer United Kingdom; 3 TReND in Africa Brighton United Kingdom; 4 Yobe State University Damaturu Nigeria; 5 University of Nairobi Nairobi Kenya; 6 University Hospital of Würzburg Würzburg Germany; 7 Menoufia University Shibin El Kom Egypt; 8 International Islamic University Malaysia Pahang Malaysia; 9 Aga Khan University Nairobi Kenya; 10 KEMRI/ Wellcome Trust Research Programme Kilifi Kenya; 11 Wellcome Trust London United Kingdom; 12 Healthy Brains Global Initiative Stockholm Sweden; 13 Natasha Walker Associates Heidelberg Germany; 14 Independent science writer Cambridge United Kingdom; 15 KEMRI Wellcome Trust Research Programme Kilifi Kenya; 16 Pwani University Kilifi Kenya; 17 University of Oxford Oxford United Kingdom; 18 University of the Witwatersrand Johannesburg South Africa

**Keywords:** Africa, scientific communities, research strategy, research capacity, mental and neurological health, impact of climate change, Child development, None

## Abstract

Working in Africa provides neuroscientists with opportunities that are not available in other continents. Populations in this region exhibit the greatest genetic diversity; they live in ecosystems with diverse flora and fauna; and they face unique stresses to brain health, including child brain health and development, due to high levels of traumatic brain injury and diseases endemic to the region. However, the neuroscience community in Africa has yet to reach its full potential. In this article we report the outcomes from a series of meetings at which the African neuroscience community came together to identify barriers and opportunities, and to discuss ways forward. This exercise resulted in the identification of six domains of distinction in African neuroscience: the diverse DNA of African populations; diverse flora, fauna and ecosystems for comparative research; child brain health and development; the impact of climate change on mental and neurological health; access to clinical populations with important conditions less prevalent in the global North; and resourcefulness in the reuse and adaption of existing technologies and resources to answer new questions. The article also outlines plans to advance the field of neuroscience in Africa in order to unlock the potential of African neuroscientists to address regional and global mental health and neurological problems.

## A unique value proposition for neuroscience

Sub-Saharan Africa is one of the last regions of accelerating population growth in the world, which means it has a relatively young population, and an extraordinary set of environmental and genetic factors have an influence on brain health in the region. Africa is also characterized by a higher incidence of infections, trauma and violence, while greater exposure to toxins and poor nutrition is set against a background of dramatically greater genetic diversity. All these factors combine to create opportunities for the neuroscience community in Africa to make breakthroughs in basic research and to develop tailored interventions to promote brain health and wellbeing in Africa.

While a high prevalence of mental, neurological and substance-use disorders challenges the African neuroscience community (Feigin et al., 2017; Feigin et al., 2019), there are opportunities too. Low doctor-to-patient ratios expose the typical clinician to more patients, increasing opportunities for developing expertise in the field. This exposure also provokes research questions that are more directly relevant to the local environment. Clinical trials become feasible over shorter time spans, with benefits not only for Africa, but also the global North. For example, recruiting patients for clinical trials in traumatic brain injury takes less time in Africa compared to Europe. Finally, there is much room for clinically-oriented neuroscience research to improve healthcare in Africa, especially in young people. Specific goals include the enhancement of developmental potential and mental health, and the reduction of mortality and neurological disability.

**Figure 1.**
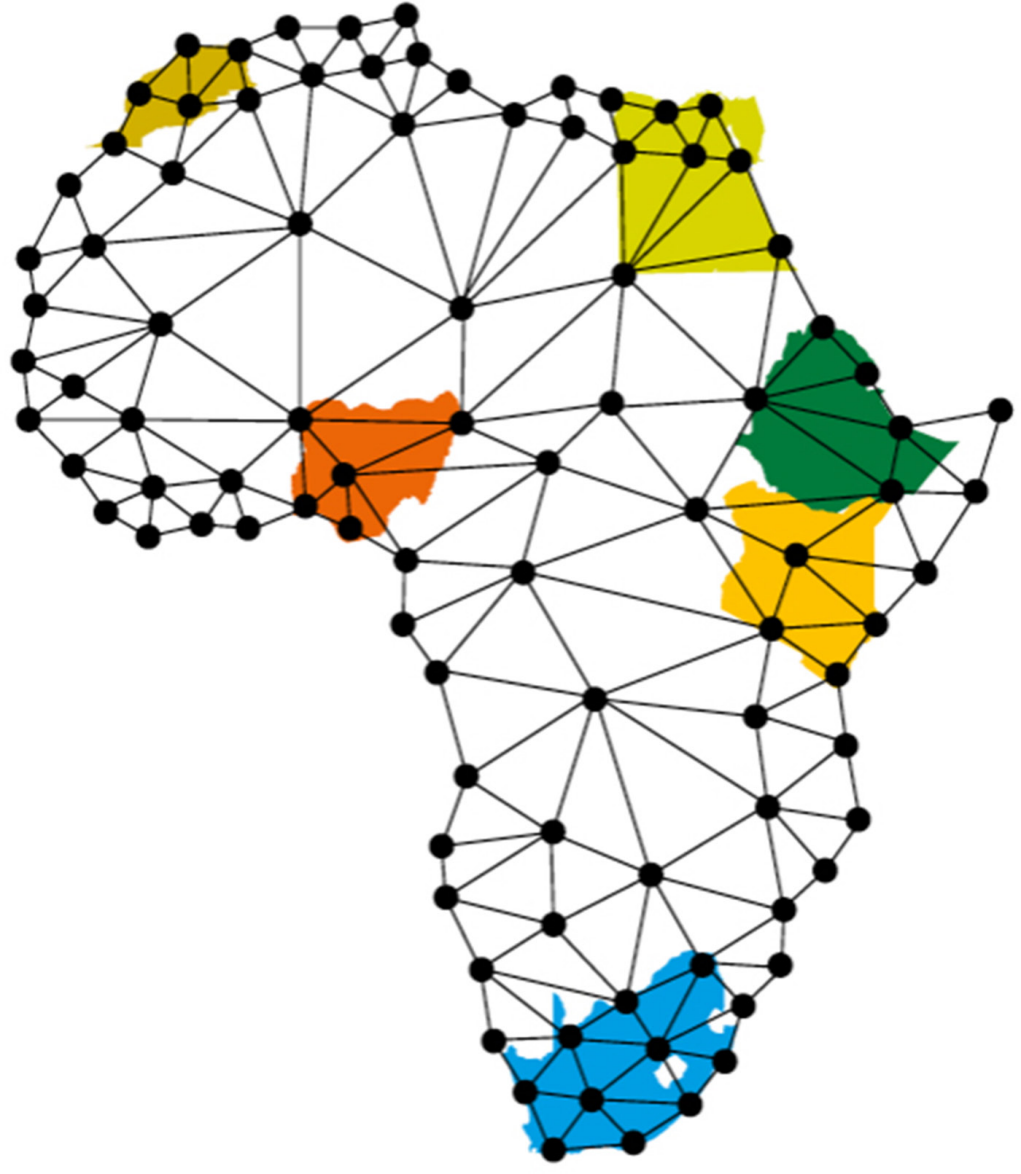
Showcasing African Neuroscience. Six African countries were represented at the two day virtual Showcasing African Neuroscience meeting in November 2021: Egypt, Ghana, Kenya, Nigeria, South Africa, Tanzania and Tunisia. An additional three half-day workshops were held in December 2021 and January 2022 to explore key themes identified at the initial meeting and to develop the strategy outlined in this article.

In this article we report on the Showcasing African Neuroscience initiative and present the outputs from meetings that were held between November 2021 and January 2022 (Figure 1). In particular, we discuss the six “domains of distinction” for the African neuroscience community that emerged from these meetings, and outline plans to help this community to fulfil its potential. A longer report will be published in the near future.

## Six domains of distinction for potential of neuroscience in Africa

### Diverse DNA of African populations

Humans evolved in Africa, and then dispersed across the world, resulting in the DNA of those of African ancestry exhibiting the greatest diversity (Campbell and Tishkoff, 2008). Given that all humans trace their genetic lineage back to Africa, there is global interest in using genomic information from African populations to study human evolution, early migration patterns and genetic architecture of health conditions.

Genomic diversity in rare variant discovery and common variant genetic risk research in neuropsychiatric or neurological conditions in African ancestry populations is beginning to shed light on risk and resilience for diseases at the regional and global level. For example, having the Apolipoprotein E allele is the strongest risk factor (after aging) for late-onset Alzheimer’s disease in the European ancestry population. However, despite a high frequency of Apolipoprotein E allele amongst those of African ancestry (Zekraoui et al., 1997), it does not appear to be as important a risk gene for Alzheimer’s disease development in this population (Farrer et al., 1997; Hendrie et al., 2014). This example demonstrates that whole-genome association studies need to have greater representation from African populations so that their findings can be generalized to global populations.

### Diverse African flora, fauna and ecosystems for comparative research

The diversity of animal models in the African ecosystem (e.g., Meriones shawi, jerboa, rabbit, baboon, monkey, buffalo, elephant, guinea pig and chameleon) is a unique strength, as it can offer novel perspectives on brain health and disease, the transmission of nervous system diseases from animals to humans, and screening for novel drugs (Maina et al., 2021; Maina et al., 2019). Research into the nervous systems of these fauna has already delivered breakthroughs in neuroscience (https://fbresearch.org/medical-advances/nobel-prizes/).

Africa’s fauna could provide access to model systems that might reveal clues to solving problems in basic and clinical neuroscience today. The naked mole rat, for example, can endure extreme hypoxic conditions, has an unusual nociceptive system, and seems not to show cognitive decline associated with aging. The spiny mouse, *Acomys*, is being used to study spinal cord regeneration, due to its ability to heal wounds with little scarring (Gaire et al., 2021). Examples such as these demonstrate the potential of novel model systems to drive scientific breakthroughs, and research in this relatively untapped area is expected to bring these – as well as new, genetically-modified model organisms – to the global stage, generating opportunities to study relevant brain disorders.

### Child brain health and development

The early months and years in a child’s life set the foundation for lifelong physical and mental health. During this stage, when nearly all physical, cognitive and social skills are being developed, the 500 million children and adolescents in sub-Saharan Africa face a unique set of challenges. Adverse events and exposures during this sensitive period can impact brain health and development throughout the course of life. While large studies have been done in the global North, less is known about the risk architecture of intellectual and developmental disabilities in African ecosystems. Prospective studies, conducted in the last decade, have shown that nutrition (including iron-deficiency anaemia), infections (e.g., HIV and malaria), environmental stimulation and interaction, and psychosocial factors may all impact brain development (Wedderburn et al., 2022). In addition, social/gender inequalities further amplify developmental differences. Building on this work, more cohorts are currently being recruited to provide opportunities for scientific discovery and for tailoring interventions to promote the best potential for child brain health and wellbeing on the continent.

One key question is how can non-invasive techniques (neuroimaging, microbiome, functional measures such as EEG, culturally appropriate developmental tools) be used in resource-limited settings to address questions around the trajectory of brain growth and development in this high-risk context? While these tools are available in limited contexts across the continent, the challenge is to take this new technology to scale in this rich, but barely explored region (Deoni et al., 2021).

### Impact of climate change, environmental risks and conflicts on neurological and mental health

While well-resourced, industrialised economies are strong enough to withstand the immediate effects of climate change, poorer regions often have to face both gradual climatic change and extreme weather events with fewer resources (and often with an inadequate energy infrastructure; Vergunst and Berry, 2021). Children are particularly sensitive, suffering from the direct effects of climate change (such as temperature instability) and a host of indirect effects such as poor air quality (burning of wood for heat and cooking), malnutrition (food insecurity from crop failures), disease (cholera outbreak following flooding), and parasites. Emerging research suggests that climatic factors, including heat stress during pregnancy, negatively impacts both maternal and new-born outcomes (Randell et al., 2020).

Collaborative and interdisciplinary work is needed to understand the potential impact of climate change on early brain development in Africa. Moreover, war and conflicts are known to influence child and adult neurological and mental health (Kong et al., 2022; Murthy and Lakshminarayana, 2006). Many parts of Africa are also affected by militarised conflicts, but the impact of these conflicts on neurological and mental health in different ancestral populations and geographical locations is not well understood. Data science has the potential to help answer questions related to the impact of climate change, environmental risks and conflicts, especially if it can take advantage of data from mobile phone networks (which are ubiquitous in Africa). Specific examples include studies of brain health at the population level, and the development of predictive models to identify potential risks to a population.

### Access to clinical populations with important conditions less prevalent in the global North

African neuroscience also has an unfortunate advantage when it comes to the study of brain injuries and infections. The region has the highest incidence of traumatic brain injury and spinal cord injury, largely caused by road traffic accidents and assaults. Brain infections are more common, caused by bacterial meningitis, tuberculous meningitis, neurocysticercosis, HIV and cerebral malaria; hydrocephalus and epilepsy are also more common given the prevalence of brain infections, injury and spina bifida as underlying conditions (Dewan et al., 2018; Hyder et al., 2007; GBD Tuberculosis Collaborators, 2018; Wright et al., 2021). While the socio-economic reality of the African environment, in addition to its relatively young population, accounts for the high prevalence of these conditions, these factors also offer opportunities for research. For example, the fact that some of these conditions share secondary mechanisms of progression should prompt research into neurobiomarkers. Further, African neuroscience can test the efficacy of therapeutics in local settings, with the aim of developing safe, appropriate and accessible treatments (Ketharanathan et al., 2019; Rohlwink et al., 2019).

### Innovative reuse and adaptation of existing technology and resources to answer new questions

Restricted access to state-of-the-art technologies has compelled African neuroscientists to find innovative approaches in pursuit of their research goals. Some have adapted existing technologies and the unique systems around them to build their own equipment (Kumbol et al., 2018; van Niekerk et al., 2019); others are developing specialised research methodologies to generate robust, culturally-relevant scientific data on diseases affecting their communities (Monteiro, 2014; Sahlu et al., 2019). Many have ventured into medicinal flora research and collaborate with the public and traditional healers to understand the scientific validity of traditional medicine and how it can result in the development of effective and affordable therapy for world diseases (Abegaz, 2016; Maina et al., 2021; Terburg et al., 2013). For example, African research on medicinal flora provides a novel avenue for discovering treatments for currently untreatable diseases, such as Alzheimer’s disease and depression.

## Plans to advance the field of neuroscience in Africa

**Figure 2.**
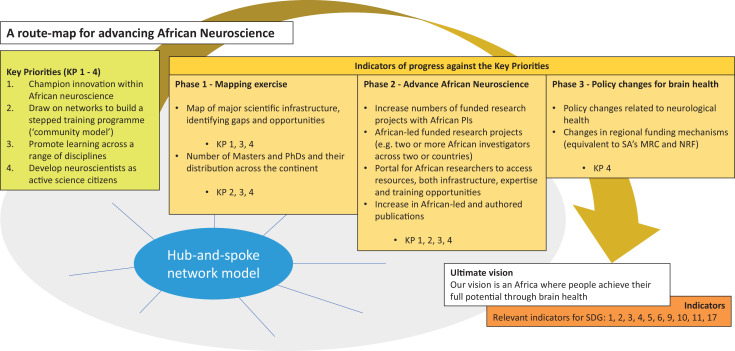
A route map for advancing African neuroscience. The overall goal of the Showcasing African Neuroscience initiative is to strengthen the neuroscience research community in Africa towards an ultimate vision of optimised neurological and mental health. There are four key priorities (left-most column), and indicators will be used to assess progress against these key priorities over three phases. The hub-and-spoke network model is a key driver of the inititiative. The project is also relevant to 10 of the 17 sustainable development goals (SDGs) set by the United Nations.

African neuroscience has yet to reach its full potential. However, existing initiatives – such as H3Africa, DSI-Africa and DELTAS Africa, interdisciplinary hubs such as the Neuroscience Institute at the University of Cape Town, and other pockets of research excellence – are coming together following the dramatic changes brought about by the COVID-19 pandemic. The Showcasing African Neuroscience project offered a ‘moment of opportunity’ to take a fresh look at neuroscience in Africa, and develop a strategy to help the African neuroscience community to fulfil its potential (see Figure 2). Four key priorities for advancing African neuroscience were identified.

### Champion innovation within African neuroscience

The meeting resolved that in order to foster innovation in African neuroscience, faculties should build on existing pockets of excellence and innovation (such as resourcefulness in the reuse and adaption of existing technologies and resources to answer new questions); cross-train local technicians to maintain the variety of systems making up local laboratories and clinics; capitalise on the commercial potential for research and develop models for export to other developing regions; and develop platforms for research collaboration and the sharing of knowledge.

### Draw on networks to build a stepped training programme (‘community model’)

Developing our neuroscience leaders of the future requires exposure to a range of techniques, settings and expertise. An effective way to achieve this is to involve different people and opportunities from across the African neuroscience community and communities of African neuroscientists in the diaspora. A community approach to training encourages an environment of cross-learning, collaboration and networking. Examples of existing networks that are supporting this area include Society for Neuroscience in Africa, African Child Neurology Association, and International Brain Research Organisation training schools.

### Promote learning across a range of disciplines

Early-stage neuroscientists recognise the importance of gaining exposure to different disciplines and skills in order to build experience and depth as scientists and mentors. Individuals should be afforded opportunities to network beyond their research specialism and institution in order to develop broader scientific understanding and to stimulate new ideas and innovative approaches. This includes opportunities to showcase progress at conferences, in professional societies, and through journals.

### Develop neuroscientists as active science citizens

It is essential for all scientists in Africa to actively participate in the broader scientific community, beyond their specific fields of study. Our early-career researchers are our future leaders. If public universities can be strengthened to support this group with funded positions that bridge the gap between graduation and independence, the development of their skills and experience will enable them to participate as torchbearers and to better engage with wider society. Demonstrating to both scientists and their institutions the importance of participating as active science citizens is essential to capture buy-in.

## The future for African neuroscience

The *Showcasing African Neuroscience* vision is to work towards a hub-and-spoke network model for neuroscientists in Africa, with networking capabilities, including a database of information about their research interests, equipment and resources they can share, and opportunities such as grants, collaborations, training and mentoring. The hub should be flexible in its structure to suit different contexts, with sub-networks where relevant. The broad aims of the hub are to: promote greater interaction, networking and collaboration within and between scientific fields, institutions, countries, and the African continent; build capacity within African neuroscience by sharing information, expertise and resources; amplify the voice of neuroscientists across the continent by advocating together on shared issues; and encourage innovative neuroscience. One such ‘virtual network’, MHIN Africa (where MHIN is short for Mental Health Innovation Network), is already in existence. More recently, the Neuroscience Institute at the University of Cape Town is a significant physical example of such an initiative, offering opportunities to draw together African neuroscience.

African neuroscience researchers rely on international funding sources, often tied to research questions that are not directly relevant to Africa. Through this project, and with the support of both international and local funding mechanisms, African neuroscience launches an ambitious, yet practical, vision for a successful and thriving African neuroscience ecosystem (Figure 2). Our vision is to achieve ground-breaking science, to support neuroscientists at every stage of their career, and to provide the infrastructure required to sustain the development of neuroscience in Africa.
